# Exploring the application of knowledge transfer to sports video data

**DOI:** 10.3389/fspor.2024.1460429

**Published:** 2025-02-07

**Authors:** Shahrokh Heidari, Gibran Zazueta, Riki Mitchell, David Arturo Soriano Valdez, Mitchell Rogers, Jiaxuan Wang, Ruigeng Wang, Marcel Noronha, Alfonso Gastelum Strozzi, Mengjie Zhang, Patrice Jean Delmas

**Affiliations:** ^1^ IVSLab, The University of Auckland, Auckland, New Zealand; ^2^ UNAM, Monterrey, Mexico; ^3^ Riki Consulting, Auckland, New Zealand; ^4^ NAO Institute, The University of Auckland, Auckland, New Zealand; ^5^ One New Zealand Warriors, Auckland, New Zealand; ^6^ Centre for Data Science and Artificial Intelligence, Victoria University of Wellington, Wellington, New Zealand

**Keywords:** artificial intelligence, computer vision, transfer learning, zero-shot learning, player re-identification, Rugby League, Netball

## Abstract

The application of Artificial Intelligence (AI) and Computer Vision (CV) in sports has generated significant interest in enhancing viewer experience through graphical overlays and predictive analytics, as well as providing valuable insights to coaches. However, more efficient methods are needed that can be applied across different sports without incurring high data annotation or model training costs. A major limitation of training deep learning models on large datasets is the significant resource requirement for reproducing results. Transfer Learning and Zero-Shot Learning (ZSL) offer promising alternatives to this approach. For example, ZSL in player re-identification (a crucial step in more complex sports behavioral analysis) involves re-identifying players in sports videos without having seen examples of those players during the training phase. This study investigates the performance of various ZSL techniques in the context of Rugby League and Netball. We focus on ZSL and player re-identification models that use feature embeddings to measure similarity between players. To support our experiments, we created two comprehensive datasets of broadcast video clips: one with nearly 35,000 frames for Rugby League and another with close to 14,000 frames for Netball, each annotated with player IDs and actions. Our approach leverages pre-trained re-identification models to extract feature embeddings for ZSL evaluation under a challenging testing environmnet. Results demonstrate that models pre-trained on sports player re-identification data outperformed those pre-trained on general person re-identification datasets. Part-based models showed particular promise in handling the challenges of dynamic sports environments, while non-part-based models struggled due to background interference.

## 1 Introduction

Interest in Artificial Intelligence (AI) and Computer Vision (CV) to transform how viewers experience sports has increased over the past decade. Various downstream tasks have benefited from these methods, including predictive analysis (1, 2) and broadcast commentary (3, 4), and have provided informative insights to help coaching staff predict or prevent injuries (5, 6). However, there is a need for more efficient methods that can be applied to any sport, regardless of the budget and viewership of the governing body. CV techniques have influenced many major sporting leagues for decades, such as MLB, where CV tools (radar guns and LiDAR scanners) have been used for 15 years to estimate game statistics such as ball velocity, spin rate, and movement as part of their Statcast system (7). Similarly, the NBA just launched Spiderverse-like cinematics using NBA-voice-trained generative AI tools to supplement basketball game experience (8) for the viewers, or in the National Football League (NFL), where graphical overlays are placed on-screen based on helmet detection models (9).

Smaller sporting leagues may be unable to compete with these experiences because of the data acquisition (e.g., 12 cameras per pitch field for Statcast) and development cost, including the time and cost required to annotate large datasets. This is also a trend within the wider area of Deep Learning, where alternative methods include transfer learning, in which models are trained on large datasets from one domain and then applied to another similar domain, potentially with fine-tuning (10, 11). This is common for object detection, in which many models are pre-trained using very large datasets, such as the MS COCO dataset (12). This is relevant to sports where downstream applications, such as performance analysis (13–16), injury prevention (5, 17), tactical analysis (18–21), video event annotation (22), and video summarization (23) to name a few, rely on simple underlying action recognition and re-identification models, which have been widely researched in the CV community.

Compared to generic video footage, videos of sporting events typically benefit from background homogeneity. The background, such as a field or court for many team sports, remains constant, with fewer distractors during the video. This simplifies distinguishing or segmenting individuals from the background compared with other applications. However, other aspects of sports make the transfer of models from other tasks challenging. An example of this is re-identification methods that may rely on the clothing worn by individuals to match individuals between observations. Re-identification in sports is particularly challenging due to the dynamic nature of the environment and the frequent occlusions that occur during gameplay. Players’ movements are rapid and often unpredictable, leading to significant variations in pose and appearance. Furthermore, while team uniforms provide some consistency, they can also introduce ambiguity as all players on a team wear similar attire, making it difficult to distinguish between them using clothing alone. Sports videos often involve multiple cameras with varying angles, resolutions, and lighting conditions, adding another layer of complexity to the re-identification task. The problem is further compounded by the fact that players frequently interact closely, which can result in partial occlusions and overlapping body parts, making it difficult to extract clear and distinct features for each player.

Another alternative method is Zero-Shot Learning (ZSL), a Machine-Learning technique that recognizes objects, categories, or instances without seeing any labeled examples of those objects during the training phase. Instead of relying on labeled training data for each specific category, ZSL leverages auxiliary information to transfer knowledge from seen classes (those with training data) to unseen classes (those without training data) (24). This approach is particularly valuable when collecting labeled data is impractical or infeasible. One specific method of ZSL that is particularly relevant to our study is the use of feature embeddings derived from pre-trained models for similarity measurement. In this approach, a model is first trained on a large dataset with labeled instances to learn rich feature representations. These learned feature embeddings capture essential characteristics and can be used to measure the similarity between new instances and known categories. For ZSL, the pre-trained model’s feature embeddings of seen classes are used to represent unseen classes by mapping them into a common embedding space. The similarity between an unseen instance and the known instances is then calculated using metrics such as Cosine Similarity or Euclidean distance.

This study investigates the effectiveness of ZSL for player re-identification in sports, using Rugby League and Netball as case studies. We test multiple existing methods to determine the effectiveness of different models pre-trained on out-of-domain datasets for our unseen datasets. In our ZSL testing environment, the re-identification models pre-trained on sports data (specifically, soccer) excelled in the task of Rugby/Netball player re-identification, while the models pre-trained on the person re-identification datasets were less effective. We also observed that part-based re-identification models outperformed the non-part-based models (regardless of the dataset content they were pre-trained on).

The rest of the paper is organized as follows: Section 2 details the principles of re-identification with a focus on sports applications, namely player re-identification. The next section describes the creation of our datasets, player IDs, action annotations, the re-identification models, and the metrics used for comparative results. Section 4 summarizes the results of our experiments. We conclude the study in Section 5.

## 2 Player re-identification

Re-identification of human characters has long been a major research area, with the first real-time approaches relying mostly on Markov Random Field models or Kalman filtering for real-time (15 to 30 frames per second) pedestrian detection and tracking for re-identification tasks (25, 26). Player re-identification, in the context of sports, is a crucial task involving recognizing and matching individual players’ observations across various video frames or camera views. This capability is vital for more complex analysis [such as player and team’s behaviour (27)]. It has become an integral part of many processing pipelines aiming to improve team and individual player performance, prevent injuries, and develop infotainment and game analysis applications, to name a few. The use of Global Positioning Systems (GPS) tags (28, 29) and an instrumented mouthguard (30) in Rugby League and Rugby Union provides readily available positioning in the field of all players and valuable information on impacts; such systems are typically too expensive to deploy outside professional leagues. GPS data is generally treated as confidential within teams and seldom shared beyond specific studies, as it offers direct access to team strategies and detailed individual player performance. Consequently, visual information captured by a single camera or multiple cameras placed around the playfield often becomes the primary available source, presenting several challenges:

• Visual Similarity: Players on the same team typically wear identical uniforms, making it difficult to distinguish between them based on appearance alone. While jersey numbers and names can help, they are often not visible in all video frames due to motion blur or occlusions.
• Occlusions and Interactions: Players frequently overlap, especially in close-contact sports, leading to partial or complete occlusions of individual players. Additionally, it becomes challenging to separate individual players from the group when many players are clustered together.
• Dynamic Movements: Rapid changes in player position due to running, jumping, or other movements cause significant variations in appearance and motion blur, reducing the clarity of the player’s features in the video frames.
• Varying Conditions: Variations in lighting conditions, whether due to indoor vs. outdoor settings or changing weather conditions, affect the visibility and clarity of players. Furthermore, differences in camera angles, zoom levels, and resolutions across different games or venues introduce variability that models must handle effectively.

Several reviews of state-of-the-art methods for player re-identification (for sports applications) have been published recently (19, 31). Current methods can be categorized as using (i) vision transformers which capture both global and local features, thus improving accuracy despite inherent motion blur and occlusions (15, 16); (ii) Convolutional Neural Networks (CNNs) for multi-object tracking across sports (20, 21); (iii) self-supervised learning targeting players with nearly identical visual features (13); (iv) and attention mechanisms for refined feature extraction (18). While the study (32) reports improved robustness and accuracy in player positioning using multiple camera views, the need for exact synchronization of 2D video feeds across the field and inherent inaccuracies in triangulation techniques may diminish the overall benefits. A promising framework integrating the above methods for various applications, e.g., live analytics and automated broadcasting commentary, is ZSL, where a given class of objects/actions is categorized without prior examples of such objects/actions being used during the machine learning training stage. This is particularly suited for sports video feeds where players do tend to appear or disappear suddenly and where actions may change in a supposedly unpredictable manner. In the case of players’ re-identification, ZSL can take advantage of a dataset where semantic (players’ respective position and actions) and visual information (in the bounding box surrounding a player) can be used to discriminate between known and unknown classes, e.g., a new player entering the field of view (16).

**Figure 1:**
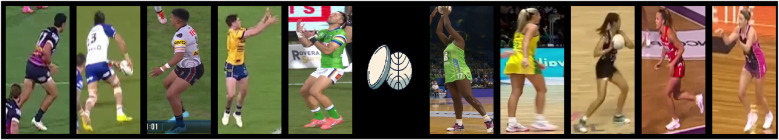
Player-crop examples in our Rugby League and Netball sub-datasets. Using these crops, we have created two re-identification datasets for ZSL evaluation purposes.

Looking at an ideal but demanding environment for testing ZSL re-identification models, we created two re-identification datasets of Rugby League and Netball broadcast videos (see Figure 1). The uniform appearance of players, frequent physical interactions leading to occlusions, rapid and varied player movements, and changing camera viewing angles contributed to the complexity of accurately identifying players’ tasks. These datasets allowed us to effectively assess the robustness of ZSL using pre-trained re-identification models (whether trained on person re-identification datasets or sports-specific datasets). This rigorous testing environment ensured that the benchmark methods/models tested were generalizable to other sports or scenarios with similar complexity. We also extended these re-identification datasets to have corresponding masked versions to analyze the models’ behavior in the presence or absence of the background for player identification purposes. By masking out the background, we aimed to isolate the player features and determine how background context influences the model’s ability to correctly re-identify players. This approach allowed us to investigate the robustness of the models further in scenarios where background information could either aid or hinder the re-identification process, providing deeper insights into model generalization across varying environments.

Before presenting the state-of-the-art methods for player re-identification, it is essential to understand the general principles of person re-identification. In general, person re-identification is critical in distributed multi-camera surveillance systems, which aims to identify if a particular individual, referred to as the query person, has been recorded in another location at a different time, either by a different camera or the same camera at a separate time. As an instance-level recognition problem, person re-identification encounters two primary challenges. Firstly, there is significant intraclass variation due to changes in camera viewing conditions. Secondly, there are minimal interclass variations, as individuals in public spaces often wear similar clothing. From a distance, as typically observed in surveillance videos, they can appear remarkably similar to Zhou et al. (33). Earlier methods primarily focused on low-level attributes, including color, shape, and local descriptors (34). In recent years, Deep Learning has revolutionized the domain of person re-identification. CNNs have become the cornerstone of this field, excelling in feature extraction through end-to-end training and diverse metric learning losses (35–37). Many approaches rely on a global strategy to tackle person re-identification, which involves learning a global representation of the individual as a single feature vector (38, 39). However, these global methods struggle with occlusion challenges due to two main reasons (40):

1. The global representation may capture misleading appearance information from surrounding objects and individuals, leading to inaccurate identification.
2. When dealing with occluded images, comparing only the visible body parts in both images is crucial. Global methods fall short in this aspect, as they utilize the same overall feature for every comparison, preventing effective part-to-part matching.

Due to the limitations of global-learning-based approaches, recent research has increasingly focused on part-based person re-identification (40–45). This approach divides the target person’s appearance into distinct parts. By focusing on specific body parts visible in both images, part-based methods can effectively mitigate the issues caused by occlusions and ensure more accurate matches.

Building on these advancements in person re-identification, its principles have also been applied to player re-identification in sports (31). When it comes to player re-identification, the challenges become even more pronounced. The dynamic and fast-paced nature of sports activities leads to frequent and severe occlusions, with players constantly moving and interacting with each other and with various elements of the playing field. For this reason, part-based person re-identification models like BPBreID (40) have been used for sports data and player re-identification purposes. For instance, PRTreID (46) is a multi-purpose part-based person representation method designed to perform role classification, team affiliation, and re-identification tasks using a single backbone. The technique employs the BPBreID model (40) with an HRNet-W32 backbone (47) to extract body-part-based features from soccer videos, incorporating additional objectives for team affiliation and role classification.

## 3 Materials and methods

### 3.1 Data collection

**Figure 2:**
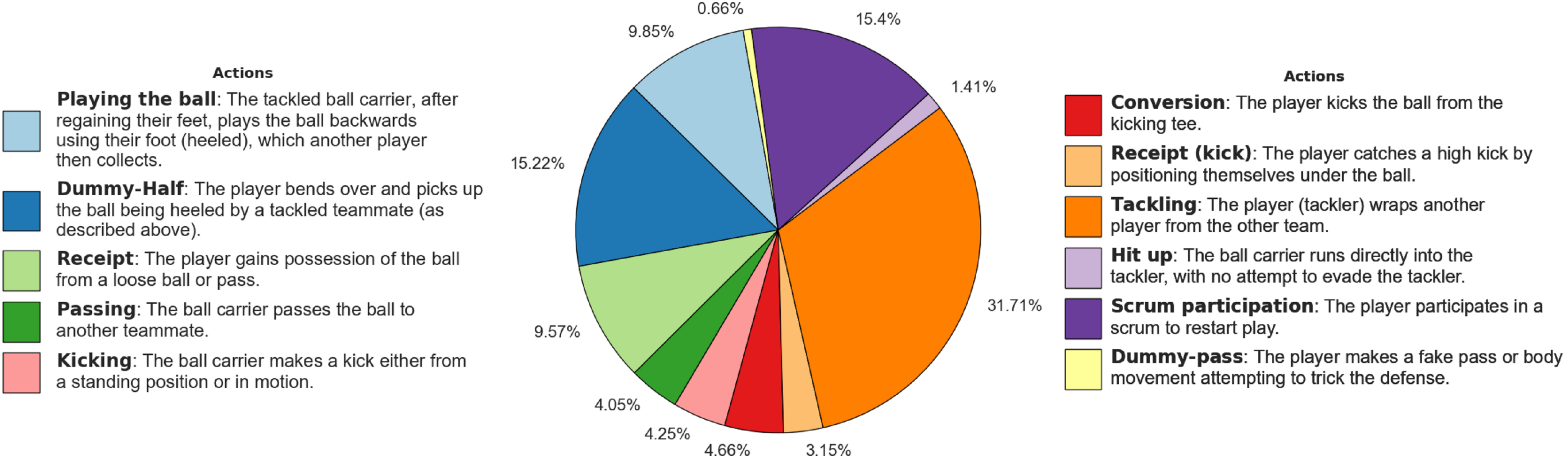
Distribution of the Rugby League player actions. The pie chart illustrates the distribution of the player actions in the dataset, with each segment representing the percentage of occurrences for each action. Action descriptions are also provided.

**Figure 3:**
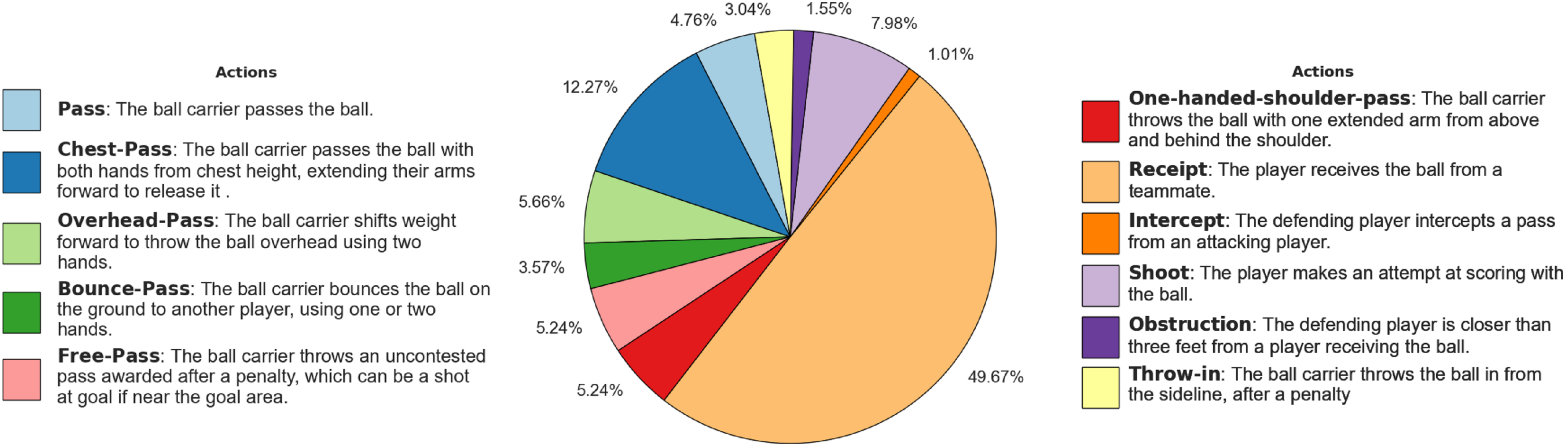
Distribution of the Netball player actions. The pie chart illustrates the distribution of the player actions in the dataset, with each segment representing the percentage of occurrences for each action. Action descriptions are also provided.

**Figure 4:**
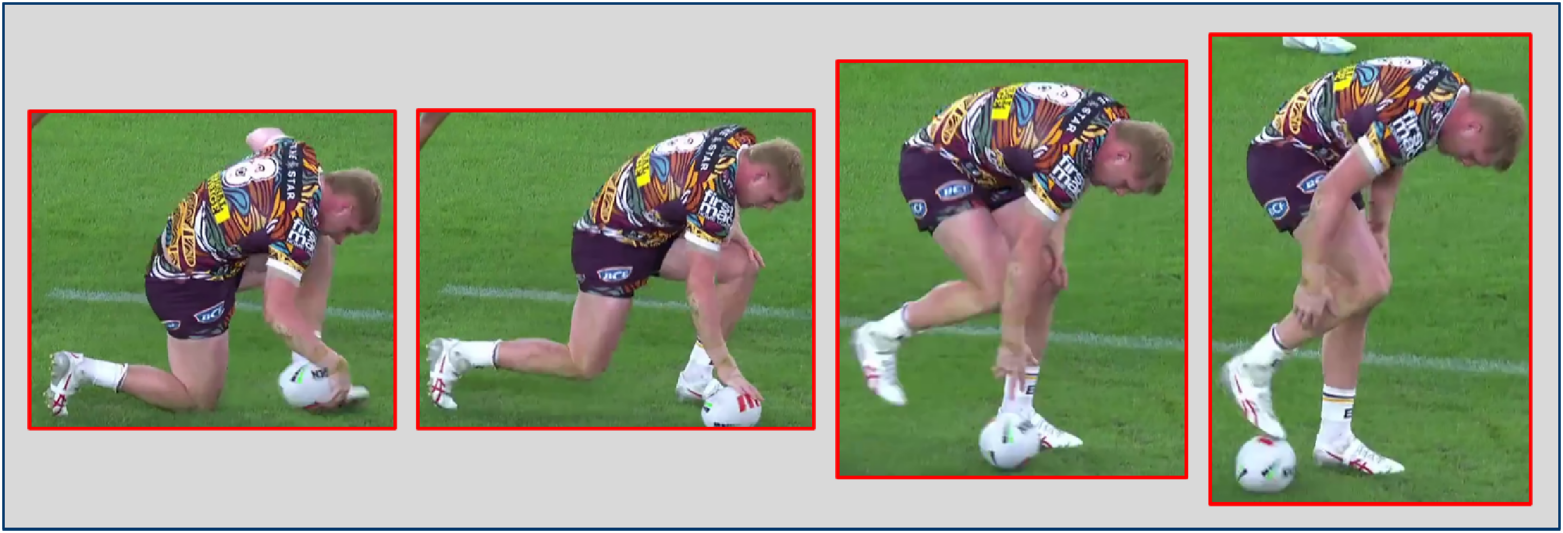
An example of the actions in the Rugby League dataset: “playing the ball” refers to when a tackled ball carrier, after regaining their feet, plays the ball backward using their foot (heeled).

We collected two datasets for professional Rugby League and Netball games to test ZSL for player re-identification, focusing on National Rugby League (NRL) games that are online (a total of 211 clips were collected for Rugby League and 152 for Netball.). We incorporated negative matches based on similar actions to create re-identification datasets encompassing many complex scenarios. By doing so, we aimed to challenge the ZSL approaches. This involves selecting negative instances where players perform the same or similar actions but are different individuals. By accessing the actions, we can ensure that the datasets include positive matches, where the same player is identified with a different action, and carefully curated negative matches with nearly the same actions, making them more challenging and realistic. As such, possible Rugby League and Netball game actions (see player’s states in Table 1 and game actions in Figures 2 and 3) were created, listing the name and textual description of the actions. For example, “*playing the ball*” in Rugby League refers to when a tackled ball carrier, after regaining their feet, plays the ball backward using their foot (heeled) (see Figure 4).

**Table 1:** Player states defined in Rugby League and Netball datasets.

Sport	State	#Annotations (%)	Description
Rugby	Stationary	27,701 (38.63%)	The player is stationary and can be in any posture as long as no motion is clear.
	In motion	36,848 (51.39%)	The player is moving at any speed, jogging, walking or sprinting.
	Being tackled	7,154 (9.98%)	The player (the ball carrier) makes contact with one or more players.
Netball	Stationary	9,367 (31.72%)	The player is stationary and can be in any posture as long as no motion is clear.
	In motion	12,090 (40.95%)	The player is in motion.
	Has ball	8,070 (27.33%)	The player is holding onto the ball.

### 3.2 Annotation

We manually annotated a total of 34,473 frames for Rugby League and 13,771 frames for Netball. On each frame, a player can described by a state and a possible action; this results in more than one annotation per frame when more than one player is involved in the action. For Rugby League data, we annotated 718 players, resulting in a total of 71,703 state annotations and 16,781 action annotations. For Netball data, we annotated 299 players, including a total of 29,527 state annotations and 1,679 action annotations. Figures 2, 3, and Table 1 contain a detailed ethogram for actions and states annotated in both datasets.

**Figure 5:**
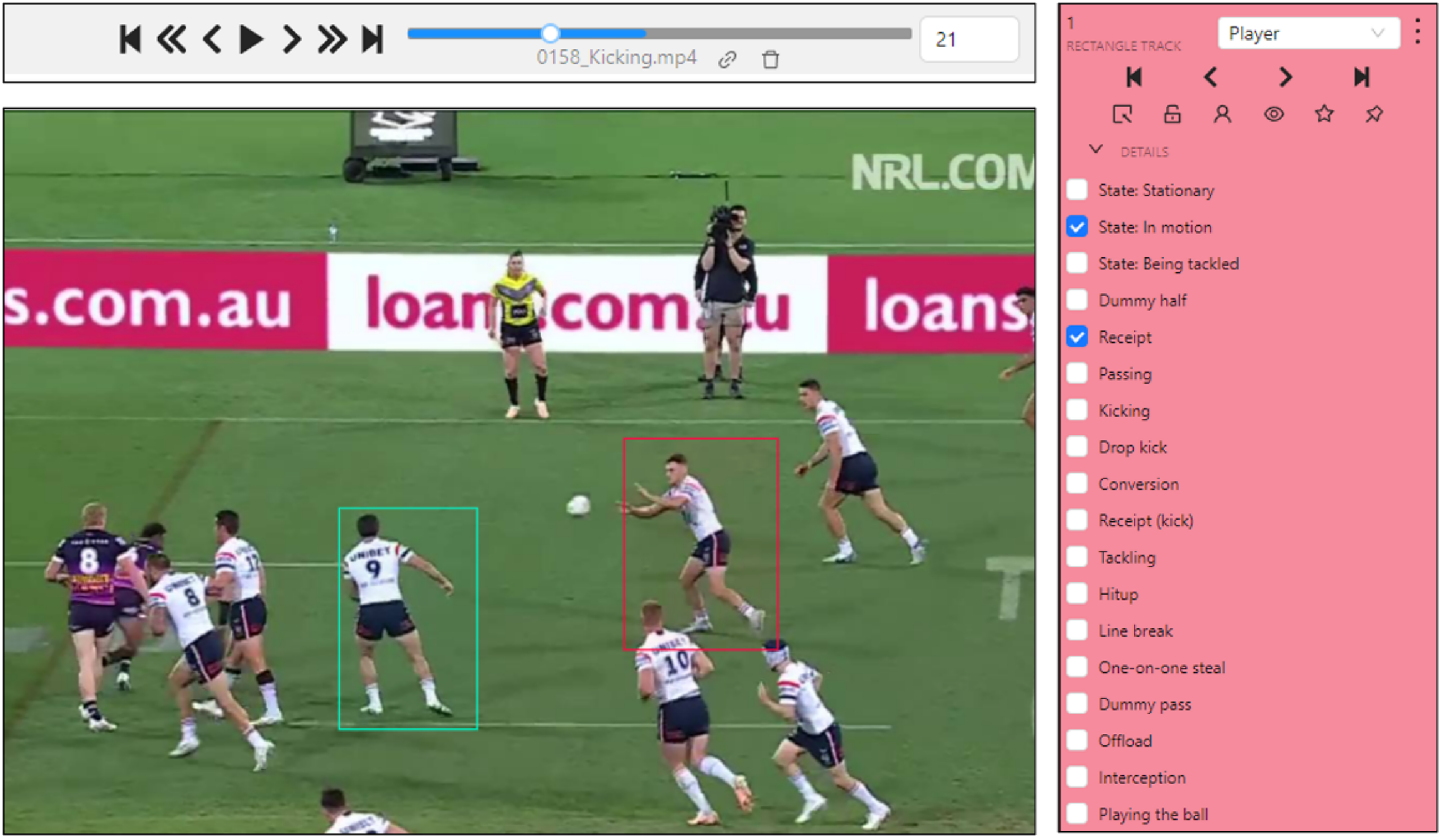
Snapshots of the annotation process on CVAT; top: annotation speed-up option, center: the current frame in the video clip, and right: The tabulated list of actions.

Five annotators labeled all videos in our dataset, typically all key players related to the actions of interest (either carrying, passing, or receiving the ball, or involved in actions that would interfere with the ball movement) in these clips using the CVAT annotation platform (48). Bounding box tracks were annotated for each player involved in the play of interest. The process could be described as selecting a bounding box around a player, typically first choosing the player carrying the ball at the start of the clip (See Figure 5 center image), and the action displayed as listed in the tabulated list of actions (see Figure 5 right image). Each player found to be interacting with the ball was added to a temporary list, and a new label, bounding box, and action were attached to them. Players were marked as occluded when they became unrecognizable, and the bounding boxes persisted through changes in camera view, common in sports feeds. Once all relevant bounding boxes to the short video clip game are labeled, a refinement process would go frame by frame through the image to adjust both bounding box size, location, and attached action. The whole process can take 10 to 30 minutes for 30 s clips or about 1,000 images.

### 3.3 Re-identification dataset

**Figure 6:**
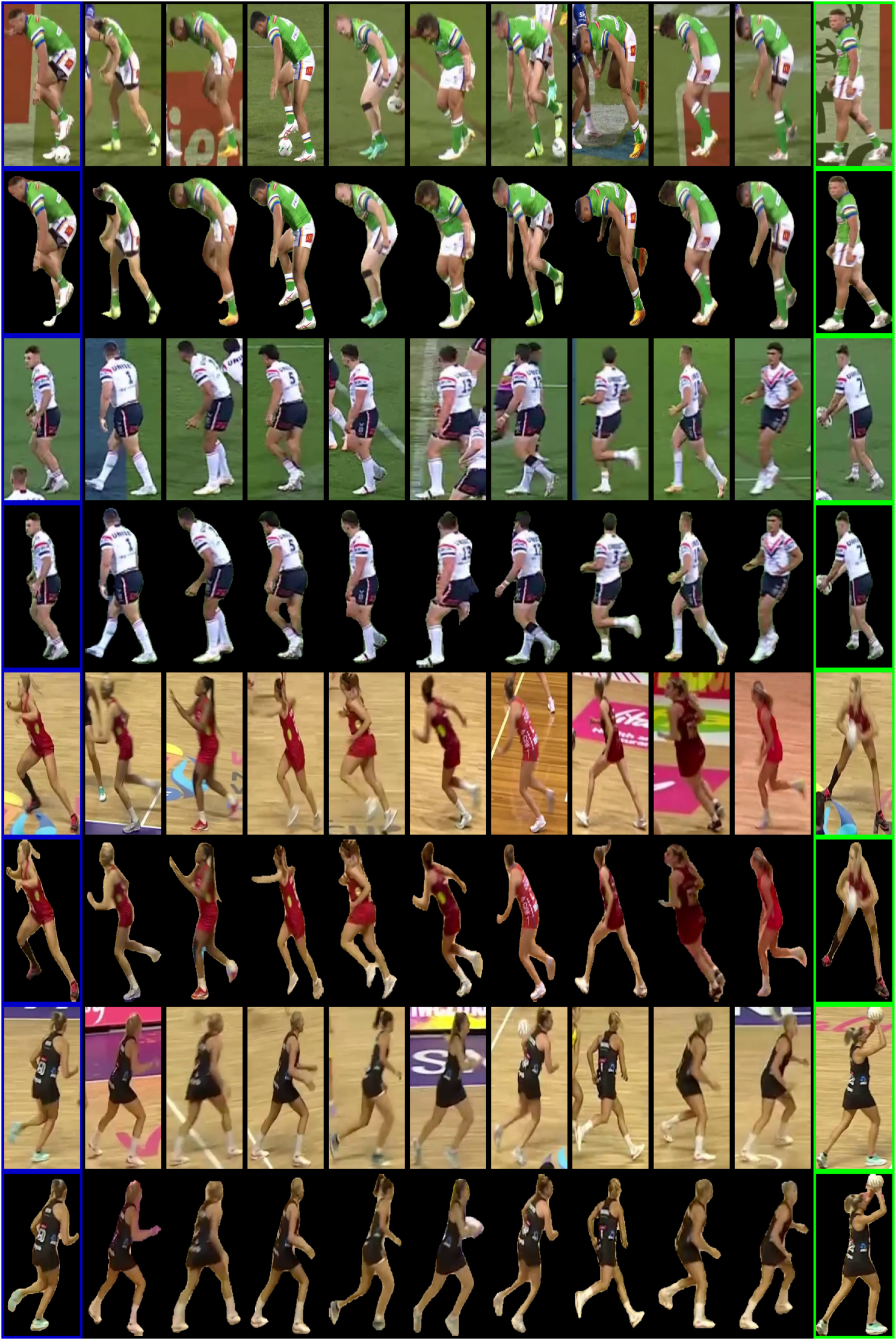
Randomly selected samples from our re-identification datasets (including their masked version). In each row, the first image is the query, the next nine player images are negative matches, and the last image shows a positive match for the selected query.

A widely adopted approach for assessing player re-identification techniques involves dividing the test dataset into two distinct parts: query images and a gallery set. This approach compares each query image against the gallery set to identify potential matches. The gallery observations are ranked based on their similarity or distance to the query image, allowing for identifying the most likely matches. This ranking process helps evaluate the effectiveness of re-identification models by determining how accurately they can match players across different images. To construct our re-identification dataset, we gathered 100 samples, each consisting of a query image paired with a gallery set of ten images. Within each gallery set, nine images represent negative matches, while one is positive. To create a challenging environment for evaluating ZSL re-identification approaches, we carefully selected negative matches where the players performed the same or similar actions and wore jerseys of similar colors to the query player. We used images of the same individual performing different actions for the positive match, ensuring that the re-identification models are rigorously tested under varied and demanding conditions. To further evaluate the behavior of pre-trained re-identification models, we also created masked versions for the Rugby League and Netball datasets. This additional analysis allows us to investigate how the models perform when the background is present or removed. Masking the background is particularly useful for evaluating re-identification models because it isolates the players, forcing the models to rely more heavily on the players’ appearance and actions rather than on contextual cues such as the field, lighting, or surrounding players. This helps determine how well the models generalize to varying environments and situations, ultimately providing a more robust assessment of their ability to focus on player-specific features. We used the bounding boxes around the players to generate the masks as inputs to a YOLOv9 (49) segmentation model. While YOLO provided a strong initial estimate for the masks, it struggled in cases where multiple players were occluded or when they were closely packed within the crop. These occlusions led to imperfect segmentation, requiring us to refine the masks in such cases to ensure accuracy manually. Figure 6 illustrates several examples from our re-identification datasets, showing both the original images and their corresponding masked versions.

### 3.4 Evaluation metrics

We used Top-k and mean Average Precision (mAP) to evaluate the performance of the re-identification models. Top-1 measures the proportion of times the correct match (i.e., the positive match) is the highest-ranked result in the gallery set. Top-3 assesses the frequency with which the correct match appears within the top 3 ranked results.Top-5 assesses the frequency with which the correct match appears within the top 5 ranked results. mAP is a summary metric that combines precision and recall to evaluate the overall performance of the re-identification model. It considers the ranking of all correct matches and provides a single score that reflects the quality of the entire ranking list.

### 3.5 Experimental design

For ZSL based on similarity, especially in re-identification, the key strategy is to map the test datasets (our re-identification datasets) into a common feature space for effective similarity measurement. We compare seven re-identification models pre-trained on person re-identification datasets and six pre-trained on sports re-identification datasets. Each dataset sample comprises a query and a gallery set. For a given query, its feature vector is compared against the feature vectors of the gallery set using Cosine Similarity. The ranking process is based on the obtained distances, with the smallest distance receiving the highest rank. The focus is applying knowledge transfer techniques to analyze sports video data, particularly Rugby League and Netball footage, using ZSL without further training. Transferring knowledge and mapping visual features to a shared feature space aims to improve the re-identification of players (in unseen video clips that were never used for training the original model). The following shows descriptions of the models used in our study.

#### 3.5.1 Person re-identification models

**MuDeep** (50): z multi-scale Deep Learning model designed for re-identification aims to learn discriminative feature representations at various scales while automatically determining the optimal scale weighting for their integration. The MuDeep network architecture is built upon a Siamese network, enhancing its ability to learn and evaluate features at different scales for effective cross-camera matching.

**HACNN** (51): it is a lightweight network architecture for jointly Deep Learning attention selection and feature representation to optimize person re-identification. This model innovatively combines the joint learning of soft pixel attention and hard regional attention with the simultaneous optimization of feature representations, specifically designed to enhance person re-identification in uncontrolled and misaligned images.

**PCB** (42): a person re-identification model to learn discriminative part-informed features. The network has two main components: (1) a part-based convolutional baseline module, which processes an input image to produce a convolutional descriptor composed of several part-level features, and (2) a refined part pooling module to address the issue of uniform partitioning, which often results in outliers within each part that are more similar to other parts. The part pooling module reassigns these outliers to their most similar parts, enhancing within-part consistency and improving the overall feature representation.

**MLFN** (52): it is a multi-level factorization network designed to learn identity-discriminative and view-invariant visual factors across multiple semantic levels. The network comprises multiple blocks, each containing several convolutional layers. The output vectors at various blocks provide compact latent semantic features at their corresponding levels. MLFN enhances performance by concatenating these multi-level semantic features into a Factor Signature feature and combining it with the final-layer deep feature, which is then subjected to a training loss.

**OSNet** (33): a person re-identification model focusing on omni-scale feature learning. The core innovation lies in its residual block design, featuring multiple convolutional streams that each capture features at distinct scales. A significant aspect of OSNet is the unified aggregation gate, which dynamically merges multi-scale features through input-dependent channel-wise weights. The architecture employs pointwise and depthwise convolutions to learn spatial-channel correlations while preventing overfitting effectively. By layering these blocks, OSNet achieves a highly lightweight structure capable of being trained from scratch on existing re-identification benchmarks.

**OSNet-AIN** (53): it is an omni-scale model capable of learning feature representations that are both discriminative (to differentiate between similar-looking individuals), and generalizable (to be used across various datasets without needing adaptation). Similar to OSNet (33), OSNet-AIN aims to capture features at multiple spatial scales and integrate them into omni-scale features. Its core building block consists of several convolutional streams, each targeting features at a specific scale. A unified aggregation gate dynamically combines these multi-scale features with channel-wise weights, enabling omni-scale feature learning. To further enhance the generalizability of feature learning, OSNet-AIN integrates instance normalization (IN) layers into OSNet, addressing cross-dataset inconsistencies.

**BPBreID** (40): a model for occluded person re-identification that employs a body part attention module and a global-local representation learning module. Using feature maps extracted from a ResNet-50 backbone (54), the Body Part attention module generates attention maps to highlight body parts, utilizing a pixel-wise part classifier trained with body part attention loss. The global-local representation learning module produces holistic and part-based features, facilitating part-to-part matching during inference.

#### 3.5.2 Sports player re-identification models

We selected six different network architectures (31) pre-trained on ImageNet and then trained/fine-tuned on the train split of the SoccerNet Re-Identification Challenge 2022 dataset (55).

**ResNet-50** (54): it consists of stacked residual blocks that use a bottleneck architecture, making it computationally efficient while still being powerful. The used model has an extra fully connected layer of 512 output channels trained for player re-identification purposes.

**OSNer-soccer** (33): it is similar to the person re-identification model discussed above but trained on the soccer data. For this reason, we rename it OSNer-soccer in this study.

**DeiT-Tiny** (56): it is based on the transformer architecture, which was originally developed for natural language processing (NLP). In DeiT, images are split into fixed-size patches (16x16 pixels), then linearly embedded and fed into the transformer as tokens. DeiT introduces a novel distillation token for knowledge distillation, where a teacher model guides the learning of the transformer. We used its smallest variant followed by dense layers to get the final feature vector for re-identification purposes.

**ViT-B** (57): ViT applies the transformer architecture to CV tasks by treating images as sequences of patches. Unlike traditional CNNs, ViT splits an image into fixed-size patches, embeds them linearly, and feeds these embeddings into a standard transformer encoder. Its base version has 12 transformer layers, 12 attention heads, and a hidden size of 768, which serves as a benchmark model. We used this architecture followed by dense layers to get the final feature vector for re-identification purposes.

**ViT-L** (57): it is a larger variant of ViT-B with 24 layers, 16 attention heads, and a hidden size of 1,024. We used this architecture followed by dense layers to get the final feature vector for re-identification purposes.

**PRTreID** (46): it is a multi-task learning model that addresses three core challenges in sports video analysis: player re-identification, team affiliation, and role classification. By integrating these tasks into a single neural network with a shared backbone, the model generates rich, multi-purpose embeddings, improving the overall performance across tasks. It uses the discussed BPBreID model (40), with HRNet-W32 backbone (58), to extract body-part-based features for players in soccer videos and adds two objectives for training the model: team affiliation and role classification.

Table 2 shows the parameter configurations for all the selected models.

**Table 2:** The parameter configurations for all the selected models.

	Model name	Input size	Output size	#Params	#Flops	model_version
Person Reid	MuDeep	256×128	1×4096	134,943,377	3,349,749,761	*mudeep*
	HACNN	160×64	1×1024	4,507,928	546,321,164	*hacnn*
	PCB	256×128	1×12288	23,508,032	4,053,270,528	*pcb_p6*
	MLFN	256×128	1×1024	32,473,024	2,771,421,376	*mlfn*
	OSNet	256×128	1×512	2,193,616	978,878,352	*osnet_x1_0*
	OSNet-AIN	256×128	1×512	2,193,616	978,878,352	*osnet_ain_x1_0*
	BPBreID	256×128	1×512	34,862,150	8,000,211,968	*bpbreid*
Player Reid	ResNet-50	256×128	1×512	24,558,144	4,054,319,616	*resnet50_fc512*
	OSNet-soccer	256×128	1×512	2,193,616	978,878,352	*osnet_x1_0*
	DeiT-Tiny	224×224	1×192	5,523,840	1,078,819,008	*deit_t_16*
	ViT-B	224×224	1×512	57,692,928	11,279,979,008	*vit_b_16*
	ViT-L	224×224	1×512	303,876,097	59,739,064,832	*deit_l_16_ls*
	PRTreID	256×128	1×512	34,862,150	8,000,211,968	*bpbreid*

#### 3.5.3 Feature extraction

The selected re-identification models were employed to extract features from our prepared Rugby League and Netball datasets for ZSL purposes. All person re-identification models were pre-trained on the Market-1501 (59) and CUHK03 (60) datasets. We utilized the Torchreid^1^ library (33, 53, 61) to build the models and load the corresponding weights, extracting features effectively. Specifically, we applied MuDeep, HACNN, PCB, MLFN, OSNet, and OSNet-AIN to obtain the corresponding feature vectors directly. For BPBreID, a part-based re-identification model, the process is more intricate as it outputs multiple feature vectors. These include holistic features ($f_{g}$, $f_{c}$, $f_{c}$) and part-based features ($\;f_{1},\ldots ,f_{k}$), where $f_{g}$ represents the global feature vector, $f_{f}$ denotes the local feature vector, $f_{c}$ is the concatenated feature vector, and k indicates the number of selected body parts (we kept the default setting, k=5). Given that $f_{g}$ and $f_{c}$ might incorporate information from occluding objects (40), we opted to concatenate $\;f_{f},f_{1},\ldots ,f_{k}$ as the final feature vector to mitigate this issue. The sports player re-identification models were also pre-trained on soccer video footage. We leveraged the repository provided by Comandur (31) on GitHub to load the weights and extract the features necessary for our analysis. Regarding the other part-based re-identification model, PRTreID, we did the same as BPBreID for the output feature vectors.

## 4 Experiments

Given the prepared Rugby League and Netball re-identification datasets, we extracted feature vectors for each player image, including query sets and their corresponding gallery sets. All experiments used Intel(R) Core(TM) i7-9800X CPU with a single NVIDIA GeForce GTX 1,660 Ti GPU. For each query feature vector, we calculated the Cosine Similarity distance to each feature vector in the corresponding gallery set and ranked them accordingly. A feature vector in the gallery set receives the highest rank if it has the minimum distance to the query feature vector, contributing to the overall Top-1 score. Furthermore, the top three and five ranked feature vectors contribute to the Top-3 and Top-5 scores, respectively. Table 3 presents the numerical evaluations for each model utilized in our ZSL approaches. We first discuss the results for each dataset separately.

**Table 3:** Numerical evaluations on the Rugby League (in green) and Netball (in yellow) re-identification datasets, including Top-k, mAP, and runtime shown by *t* per each sample (the query and gallery set). The runtimes exclude the time needed to load the models.

	Model name	Top-1 (%)		Top-3 (%)		Top-5 (%)		mAP (%)		t (s)	
Person Reid	MuDeep	23	31	46	46	62	62	40.32	46.01	0.195	0.181
	HACNN	30	40	52	54	65	72	47.08	54.18	0.176	0.182
	PCB	36	29	59	52	69	67	51.81	46.36	0.144	0.126
	MLFN	23	34	52	63	68	78	43.49	53.37	0.259	0.241
	OSNet	22	21	47	54	64	62	40.41	41.70	0.258	0.240
	OSNet-AIN	30	25	42	50	65	66	44.25	43.26	0.284	0.265
	BPBreID	47	40	71	65	85	80	62.76	56.52	0.113	0.134
Player Reid	ResNet-50	48	32	70	52	78	68	62.30	47.86	0.090	0.112
	OSNet-soccer	48	30	67	59	78	70	61.36	48.46	0.091	0.043
	DeiT-Tiny	42	25	75	51	84	65	61.06	43.38	0.152	0.110
	ViT-B	42	25	65	49	76	58	57.69	42.33	0.245	0.244
	ViT-L	54	40	76	57	84	67	67.01	54.37	0.522	0.439
	PRTreID	50	47	81	68	84	81	65.81	61.51	0.128	0.104

• **Rugby League**: regarding the Top-1 scores, the ViT-L model stands out with the highest score of 54%, indicating its capability for accurate initial re-identification compared to the other models. Close contenders are PRTreID, OSNet-soccer, and ResNet-5 models with scores of 50%, 48%, and 48%, respectively. On the lower end, OSNet has the lowest Top-1 score of 22%, making it the least effective model in this evaluation. We have almost the same trends for the other scores: Top-3, Top-5, and mAP. Overall, the results show that the re-identification models pre-trained on soccer data excelled in the task ZSL Rugby League re-identification. In contrast, the pre-trained models on the person re-identification datasets were less effective (except BPBreID, a part-based re-identification model).
• **Netball**: the PRTreID model emerged with the highest performance across all metrics, achieving the best scores for Top-1 (47%), Top-3 (68%), Top-5 (81%), and mAP (62%). Following closely, the BPBreID model also demonstrated strong results. This indicates that part-based re-identification models outperformed other models for the Netball dataset.

Considering the Rugby League and Netball datasets, several key observations emerge. For the Rugby dataset, non-part-based re-identification models like ViT-L, OSNet-soccer, and ResNet50 performed better compared to when they were tested on the Netball dataset. This could be attributed to the fact that these models were pre-trained on soccer data, which shares similarities with Rugby League regarding background, allowing the models to utilize background features for similarity measurements. However, part-based methods did not perform significantly on the Rugby dataset, as we excluded the background features for the ZSL and similarity measurements. This trend aligns well with the results from the Netball dataset, where the background differs significantly from soccer. As a result, the ZSL based on non-part-based models could not benefit from background similarity, and therefore, part-based models outperformed them on the Netball dataset.

**Figure 7:**
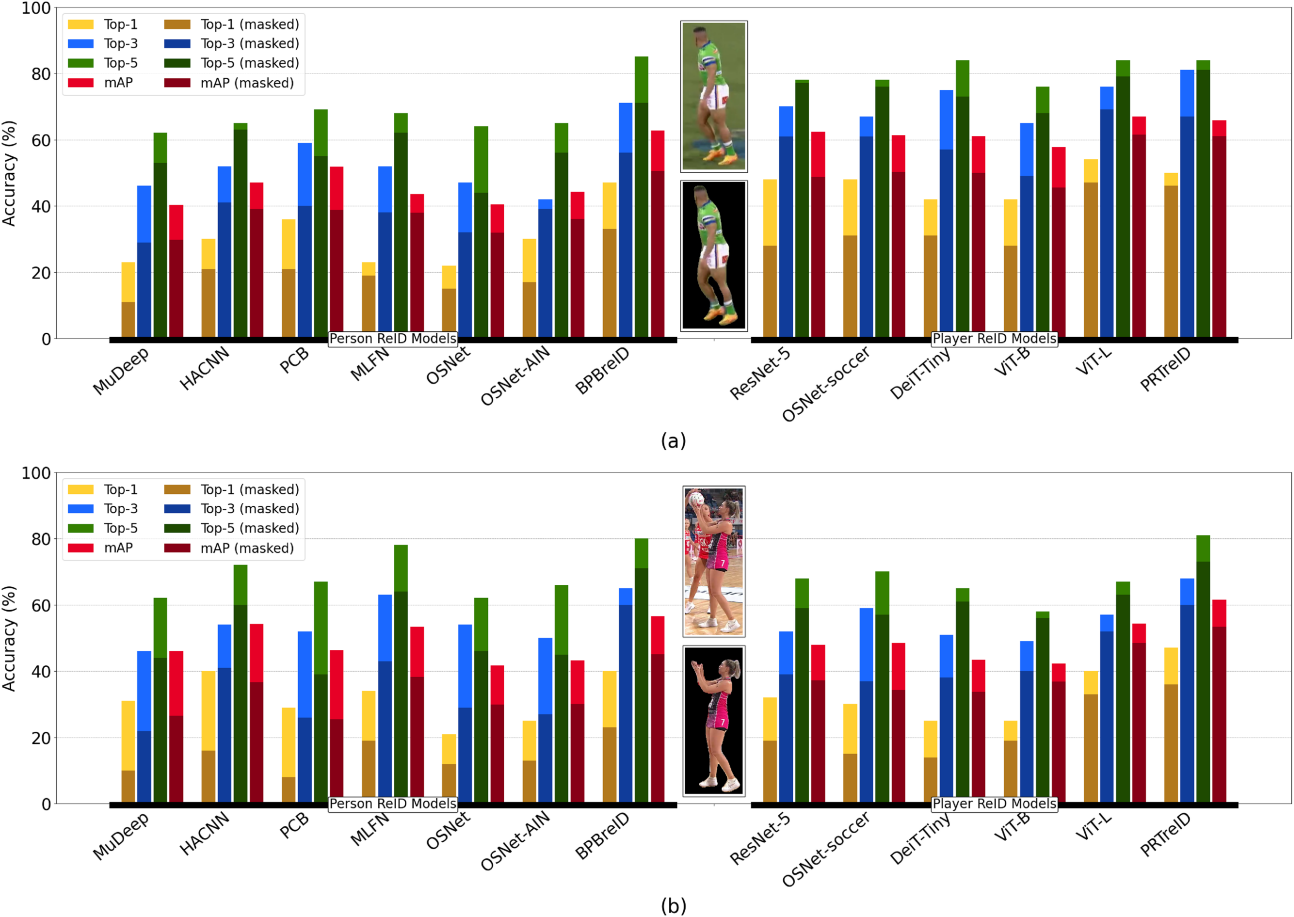
Comparasion between out selected ZLR approaches in terms of Top-k and mAP, where k∈{1,3,5}, on **(a)** Rugby League and **(b)** Netball datasets.

To further support the above discussion and analyze the behavior of the models in both the presence and absence of background features, we conducted additional tests using the masked versions of our datasets. By excluding the background, we can better evaluate how each model responded to features specifically related to the players. This additional analysis provides deeper insights into the strength of part-based vs. non-part-based ZSL methods across two different sports. Figure 7 illustrates the comparisons. The same trend was observed across both part-based and non-part-based models: removing the background and providing only the masked players to the models reduced the performance of the ZSL approaches. This reduction was particularly significant for non-part-based models trained on person re-identification datasets. This outcome suggests that these models were not focused solely on the players but also extracted features from the background. Consequently, when the features were compared for similarity measurements, they all had black-background-related features, reducing accuracy in identifying positive matches. Part-based methods, however, were less affected by this issue, as in the ZSL approach, we explicitly excluded background features for the similarity measurements. Despite this, the decrease in similarity scores for the part-based models may be attributed to their training on different re-identification datasets, which could still introduce a level of inconsistency when applied to these specific sports datasets.

**Figure 8:**
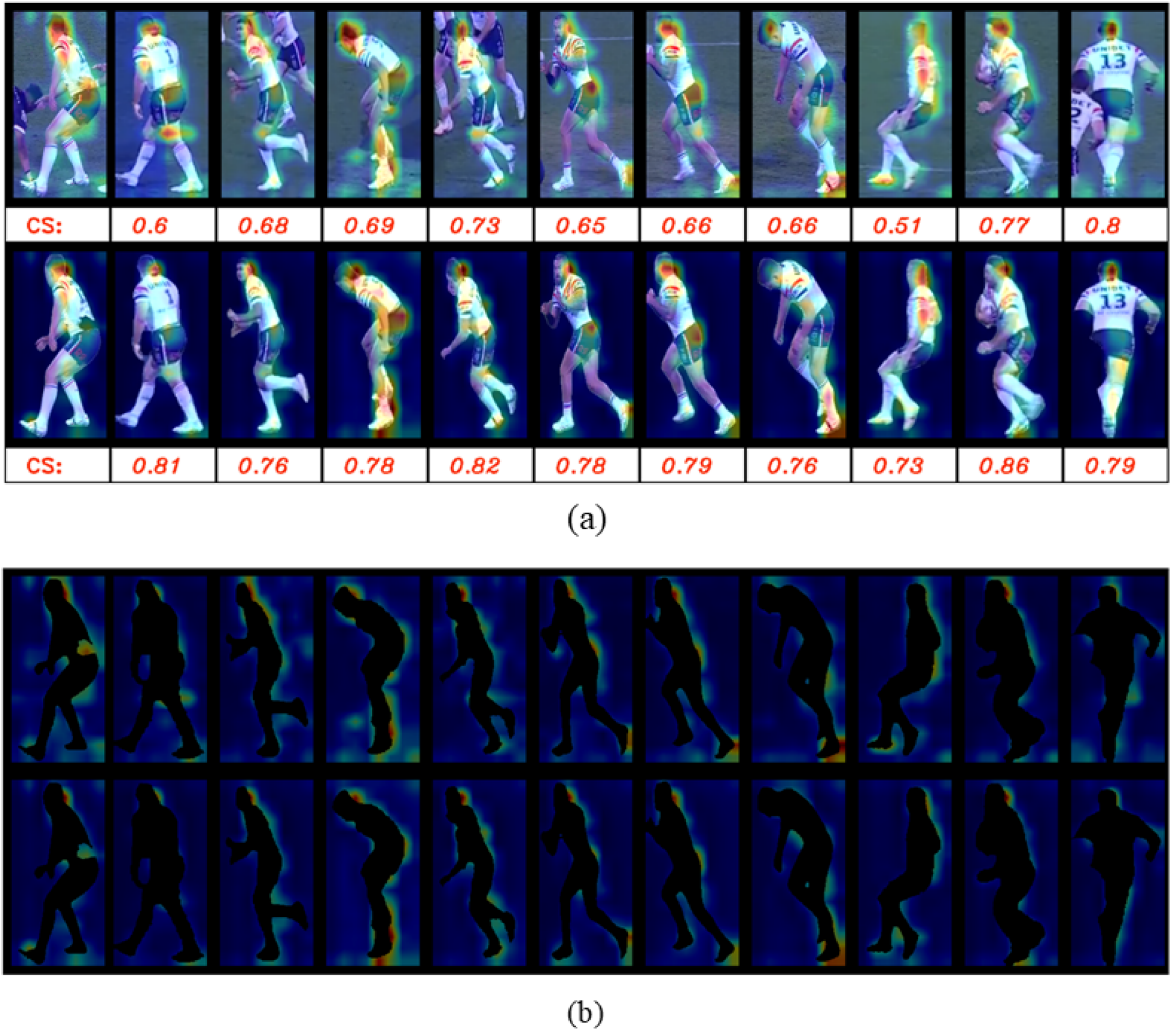
The ResNet50 heat maps on a randomly selected sample from the re-identification Rugby League dataset. Each row shows heatmaps on the player crops, from left to right: the query, nine negative matches, and positive match, where **(a)** shows the heatmaps on the original and masked player crops in the first and second rows, respectively and **(b)** shows the heat maps only for the backgrounds where first row belongs to the original crops and the second row is related to the masked version. In **(a)**, “CS” stands for Cosine Similarity, and the scores for the corresponding player and the query are shown under the player crops.

We selected ResNet-50 from our benchmark models for further analysis, primarily due to its suitability as a non-part-based re-identification model and pre-training on soccer data. Specifically, we applied the model to a randomly selected sample from the Rugby League re-identification dataset to investigate the impact of background features on the model’s attention. This was done by analyzing the attention maps generated just before the final dense layers in both the presence and absence of background pixels. Figure 8 illustrates the results. In Figure 8a, the selected sample (including both the query and gallery set) is presented with and without background, alongside their corresponding attention maps. Figure 8b further isolates the background heat maps for the masked and unmasked versions to facilitate a more explicit comparison. The findings reveal that, even when background features are removed, the model continues to focus on areas that align with background information. This behavior can be attributed to how the model was pre-trained, where background-related features were inherently integrated into the final output embeddings.

Given the challenging testing environment we established for ZSL evaluations, our findings suggest that pre-trained, non-part-based re-identification models may inadvertently incorporate background features into the final embedding. This is problematic in sports datasets, where players often share similar or identical backgrounds, such as fields or courts. This unintended background “leakage” can undermine the model’s ability to accurately distinguish between players, as it can artificially inflate similarity scores based on irrelevant environmental cues rather than player-specific features. This is where part-based re-identification models (such as BPBreID and PRTreID) demonstrate their superiority. These models are designed to decompose the input into distinct regions or parts, allowing the final embedding to separate foreground (player-specific) features from background information, which makes them the best choice for ZSL re-identification purposes. Moreover, our results highlight the potential of these models for fine-tuning in a Few-Shot Learning (FSL) paradigm. The FSL model is fine-tuned with only a few labeled samples, leveraging prior knowledge to generalize effectively with limited data. The ability of part-based re-identification models to capture more discriminative, player-specific features while minimizing background noise makes them highly effective for FSL, where limited training data amplifies the importance of relevant feature extraction.

## 5 Conclusion

In this study, we annotated two sports-specific datasets for Rugby League and Netball, containing 34,473 frames and 13,771 frames, respectively. Each frame was meticulously annotated with player IDs, actions, and bounding boxes for players involved in the action. To support the rigorous evaluation of ZSL techniques, we created two sub-datasets with standard and masked versions to focus on player re-identification under challenging conditions (we used the annotated actions to create a challenging testing environment). These conditions include visual similarities between players, frequent occlusions, dynamic movements, and varying lighting and camera angles, making the datasets ideal for ZSL analysis. A range of pre-trained re-identification models, including both CNNs and Vision Transformers architectures, were selected for evaluation. These models included part-based and non-part-based methods, pre-trained on two datasets, including person re-identification and sports player re-identification data. The results demonstrated that part-based models are promising to improve ZSL and Transfer Learning performance in player re-identification tasks, particularly for sports video data. They could also be highly effective for Few-Shot Learning (FSL), as they allow for more precise feature extraction from limited data, reducing the reliance on background information. In contrast, non-part-based models struggled due to background leakage, where background elements, often shared across players, were mistakenly incorporated into the final embeddings, skewing the model’s performance.

Potential future improvements include expanding player labeling to capture off-ball movements and strategic positioning, which may contribute to team tactics, even when players are not directly involved in the action. Additionally, the datasets and re-identification methods, particularly ZSL, could serve as a foundation for Zero-Shot Action Recognition, which will be the focus of our future work.

## Data Availability

The datasets presented in this study can be found in online repositories. The names of the repository/repositories and accession number(s) can be found below: github.com/shahrokh1106/ZSL-Player-Re-Identification.

## Footnotes

^1^Torchreid is a library for deep-learning person re-identification, written in PyTorch.
